# Amide proton transfer imaging for the determination of human papillomavirus status in patients with oropharyngeal squamous cell carcinoma

**DOI:** 10.1097/MD.0000000000029457

**Published:** 2022-07-15

**Authors:** Noriyuki Fujima, Yukie Shimizu, Masami Yoneyama, Junichi Nakagawa, Hiroyuki Kameda, Taisuke Harada, Seijiro Hamada, Takayoshi Suzuki, Nayuta Tsushima, Satoshi Kano, Akihiro Homma, Kohsuke Kudo

**Affiliations:** a Department of Diagnostic and Interventional Radiology, Hokkaido University Hospital, Sapporo, Japan; b Department of Diagnostic Imaging, Faculty of Medicine and Graduate School of Medicine, Hokkaido University, Sapporo, Japan; c Department of Advanced Diagnostic Imaging Development, Faculty of Medicine and Graduate School of Medicine, Hokkaido University, Sapporo, Japan; d Philips Japan, Tokyo, Japan; e Department of Otolaryngology-Head and Neck Surgery, Faculty of Medicine and Graduate School of Medicine, Hokkaido University, Sapporo, Japan; f The Global Station for Quantum Medical Science and Engineering, Global Institution for Collaborative Research and Education, Sapporo, Japan.

## Abstract

The aim of this study was to investigate the utility of amide proton transfer (APT) imaging for the determination of human papillomavirus (HPV) status in patients with oropharyngeal squamous cell carcinoma (SCC). Thirty-one patients with oropharyngeal SCC were retrospectively evaluated. All patients underwent amide proton transfer imaging using a 3T magnetic resonance (MR) unit. Patients were divided into HPV-positive and -negative groups depending on the pathological findings in their primary tumor. In APT imaging, the primary tumor was delineated with a polygonal region of interest (ROI). Signal information in the ROI was used to calculate the mean, standard deviation (SD) and coefficient of variant (CV) of the APT signals (APT mean, APT SD, and APT CV, respectively). The value of APT CV in the HPV-positive group (0.43 ± 0.04) was significantly lower than that in the HPV-negative group (0.48 ± 0.04) (*P* = .01). There was no significant difference in APT mean (*P* = .82) or APT SD (*P* = .13) between the HPV-positive and -negative groups. Receiver operating characteristic (ROC) curve analysis of APT CV had a sensitivity of 0.75, specificity of 0.8, positive predictive value of 0.75, negative predictive value of 0.8, accuracy of 0.77 and area under the curve (AUC) of 0.8. The APT signal in the HPV-negative group was considered heterogeneous compared to the HPV-positive group. This information might be useful for the determination of HPV status in patients with oropharyngeal SCC.

## 1. Introduction

Human papillomavirus (HPV) status in oropharyngeal squamous cell carcinoma (SCC) is an important factor impacting disease epidemiology and biological behavior. In particular, the outcome of chemoradiation therapy in patients with oropharyngeal SCC reportedly differed depending on patient HPV status, with HPV-positive oropharyngeal SCC patients tending to have a better prognosis.^[1]^ Notably, the 8th edition of the American Joint Committee on Cancer (AJCC) TNM staging system divides oropharyngeal SCCs into HPV-positive and HPV-negative tumors in their T-staging.

Differences in imaging characteristics between HPV-positive and -negative oropharyngeal SCCs have been described in several reports. On conventional computed tomography (CT) and magnetic resonance imaging (MRI), HPV-positive SCCs were shown to be more likely to have a well-circumscribed shape with intratumoral homogeneity, whereas HPV-negative SCCs tend to have unclear margins and invade nearby muscle tissue.^[2]^ HPV-positive SCCs were reported to have lower apparent diffusion coefficient (ADC) in diffusion-weighted imaging (DWI); in addition, the distribution of intratumoral ADCs was more homogeneous in HPV-positive than -negative oropharyngeal SCCs.^[3–7]^ The arterial spin labeling technique was also reported to be useful in the determination of HPV status; heterogeneous intratumoral blood flow was observed in HPV-negative oropharyngeal SCC.^[8]^ These imaging characteristics of HPV-positive/-negative oropharyngeal SCCs will be a useful supportive tool for clinical assessment.

In recent decades, amide proton transfer (APT) imaging has been increasingly used to visualize tumors including head and neck lesions.^[9–16]^ APT imaging is introduced to depict amide protons in tumor proteins and peptides. APT imaging is acquired utilizing the chemical exchange processes between free water and mobile amide protons of intratumoral proteins and peptides; this procedure is achieved with the use of the chemical exchange saturation transfer (CEST) technique.^[17]^ In APT imaging, selective saturation of amide protons is achieved by a specific radiofrequency pulse. Saturated amide protons and protons in bulk water are respectively exchanged with each other; finally, the decrease in the signal information from water protons indicates the amount of exchange for amide protons.^[18]^

To the best of our knowledge, no previous report has investigated the utility of APT imaging for the evaluation of HPV-positive or -negative oropharyngeal SCCs. Therefore, the aim of the present study was to investigate the imaging characteristics of APT for oropharyngeal SCCs and their differences in HPV-positive and -negative cases.

## 2. Materials and Methods

### 2.1. Patients

The protocol of this retrospective study was approved by our institutional review board, and written informed consent was waived. We selected the cases of 47 patients with oropharyngeal SCC who were referred to our hospital and underwent pretreatment MR scanning during the period from December 2017 to October 2020. Among them, 12 patients were excluded for lacking the data of APT images in their pretreatment MRI. Four patients whose T-stage was T1 were also excluded because their primary site was too small to evaluate in image analysis. Finally, total of 31 patients were considered eligible for the current study. All patients fulfilled the following inclusion criteria: (1) the patient was diagnosed histopathologically for the first time as having oropharyngeal SCC (not a recurrent case); (2) MRI sequences including APT imaging were performed before any treatment; and (3) information of human papillomavirus status (positive or negative) was available in the pathology record. Patients with a severe metal or motion artifact that seriously affected the image quality of the primary lesion were excluded. All patients were divided into positive or negative HPV status depending on the description in their pathology records.

### 2.2. Imaging protocol and postprocessing

All scanning was performed using a 3.0-Tesla MR unit (Achieva TX; Philips Healthcare, Best, Netherlands) with a 16-channel neurovascular coil. MRI including the APT imaging was performed to evaluate the primary tumor. APT images were acquired using a 3D turbo spin-echo sequence with spectral attenuation with inversion recovery (SPAIR) fat suppression. Volume shimming was performed before the acquisition. For the determination of the location in the placing axial plane, T1WI and Fs-T2WI were used as references. In the acquisition of the z-spectrum, imaging was performed using a total of 11 saturation points from positive and negative offsets (range: − 6.0 to + 6.0 ppm). The offset far from the water resonant frequency (− 1560 ppm) was also acquired for signal normalization. The saturation pulse strength was 2 μT and the duration was 2 seconds. Other imaging parameters were set as follows: repetition time 5637 milliseconds, echo time 6.9 msec, field of view 230 × 230 mm, acquired image matrix 96 × 96, section thickness 5 mm, echo train length 45, and sensitivity encoding factor 2; images were reconstructed as 128 × 128 with a zero-filling technique. The total imaging time was 5 minutes 11 seconds.

In addition, axial T1 weighted images (T1WI) and fat-suppressed T2 weighted images (Fs-T2WI) were also acquired with following parameter setting: (a) T1WI; a spin-echo sequence, TR 450 msec, TE 10 milliseconds, FOV, 240 × 240 mm, 512 × 512 matrix, slice thickness 5 mm, and (b) Fs = T2WI, a turbo spin-echo sequence, TR 4500 milliseconds, TE 70 milliseconds, TSE factor 9, FOV, 240 × 240 mm, 512 × 512 matrix, slice thickness 5 mm.

### 2.3. Image analysis

**Figure 1.**
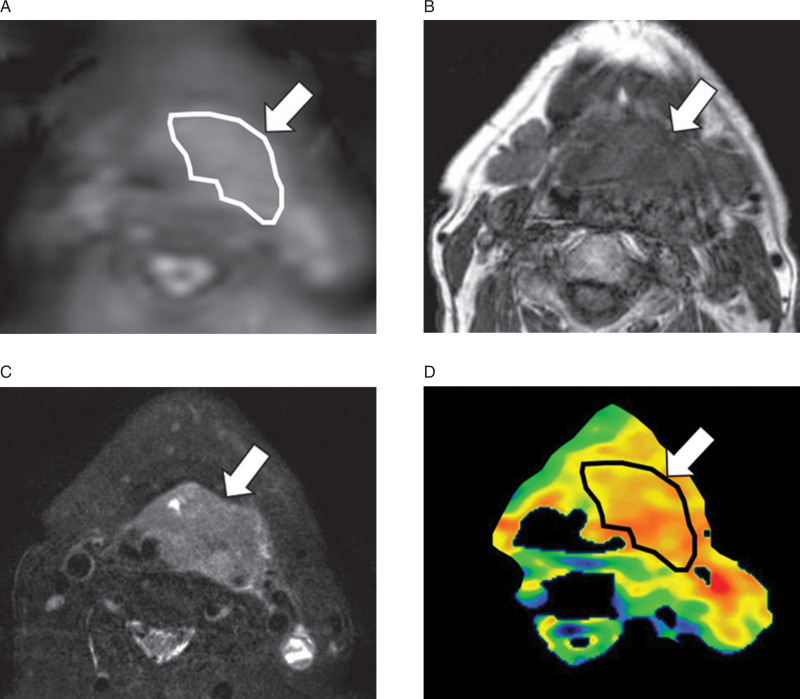
Tumor ROI delineation. To delineate each primary tumor, a polygonal region of interest (ROI) was placed on APT raw images (i.e., fast spin echo-based sequence before image processing for the calculation of the APT signal) (A: arrow). The axial T1WI (B: arrow) and Fs-T2WI (C: arrow) were used as references for ROI delineation. This ROI was copied onto the postprocessed APT images (D: arrow) for the calculation of APT mean, APT SD and APT CV.

All APT-weighted images were transferred and analyzed in the MR console. The APT images were obtained by calculating the magnetization transfer ratio asymmetry by the following process: first, the signal difference between +3.5 ppm and −3.5 ppm was calculated; thereafter, this signal difference was divided by the reference signal, which was obtained at −1560 ppm.^[19]^ A board-certified neuroradiologist with 15 years of experience in head and neck radiology, blinded to the pathology record (i.e., diagnosis of the HPV status), delineated each tumor with a polygonal region of interest (ROI) on a raw APT-weighted image dataset (i.e., APT images before the analytical process). The axial T1WI and Fs-T2WI were used as references for ROI delineation. Any area which was suspected of being a necrotic or cystic lesion was excluded from the ROI. If the tumor extended into 2 or more slices, the slice in which the largest area of tumor was depicted was selected. Thereafter, the tumor ROI was copied on the APT image (i.e., APT images after the analytical process). The APT value in each tumor was respectively determined as the mean value in the ROI. In addition, standard deviation (SD) and coefficient of variation (CV) were also calculated using the APT values from all pixels in the ROI. An example of ROI placement is shown in Fig. 1.

### 2.4. Statistical analysis

The mean APT value, the APT SD and the APT CV in the ROI were respectively compared between patient groups with HPV-positive and -negative SCCs by the Mann–Whitney U test. In addition, if significant differences were observed, parameters were further assessed using receiver operating characteristic (ROC) curves constructed for calculating the area under the curve (AUC). The sensitivity, specificity, positive predictive value (PPV), negative predictive value (NPV) and diagnostic accuracy were respectively calculated with the optimal cutoff value determined by the Youden Index in the ROC curve analysis. *P* values < 0.05 were considered significant. SPSS software (IBM, Armonk, NY) was used for all statistical analyses.

## 3. Results

Of all 31 SCC patients, 16 were classified as HPV-positive whereas 15 patients were HPV-negative. Detailed patient characteristics of the 2 groups are provided in Table 1.

**Table 1:** Patient characteristics (n = 31).

	HPV positive (n = 16)	HPV negative (n = 15)
Age		
Range	42–85	40–85
Average	62.3	67.1
Gender		
Male	16	13
Female	0	2
Subsites		
Tonsil	14	11
Base of tongue	2	2
Soft palate	0	0
Posterior wall	0	2
T-stage		
T1	0	0
T2	7	5
T3	7	7
T4	2	3
N-stage		
N0	6	3
N1	5	5
N2	5	6
N3	0	1

**Figure 2.**
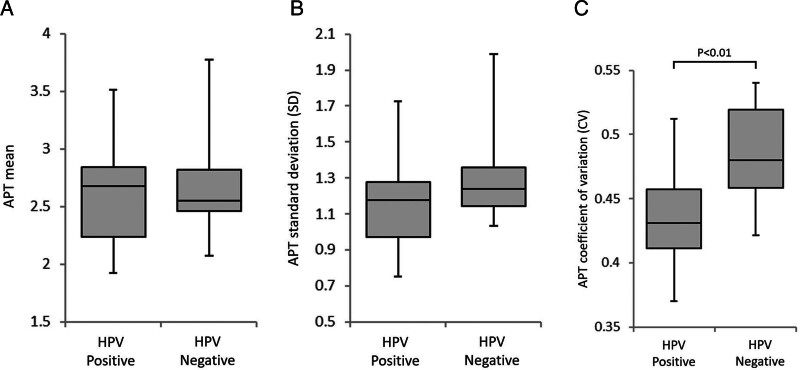
All parameters between the HPV positive and negative group. Box-and-whisker plots of APT mean (A), APT SD (B), and APT CV (C) comparing HPV-positive and -negative groups are shown. A significant difference between the HPV-positive and -negative groups was observed in APT CV (C: *P = .01).

**Figure 3.**
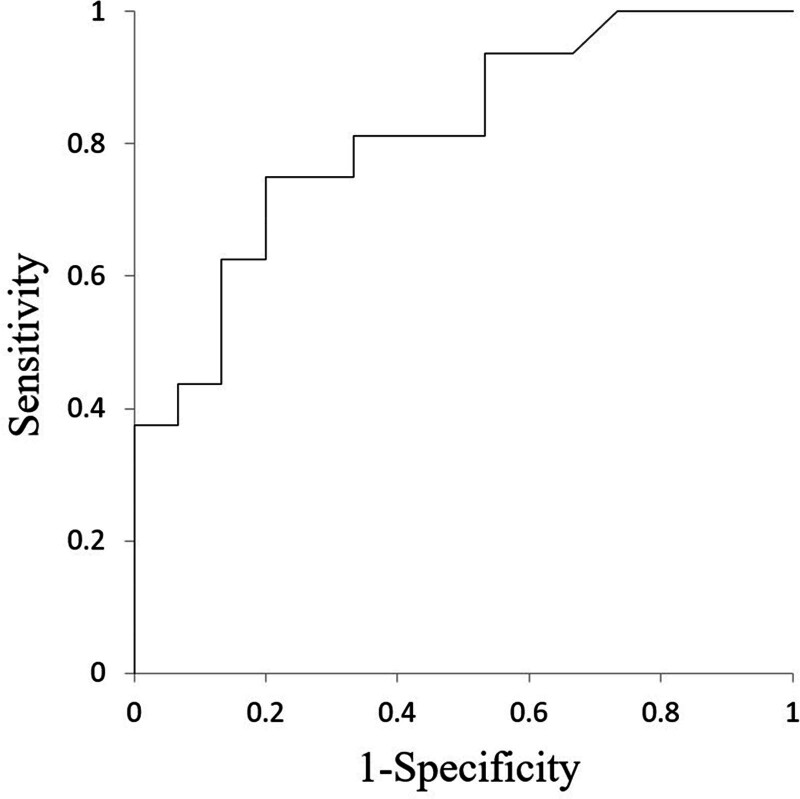
Result of ROC curve analyses. ROC curve obtained by APT CV for the determination of HPV status is shown. An AUC of 0.8 was obtained.

**Figure 4.**
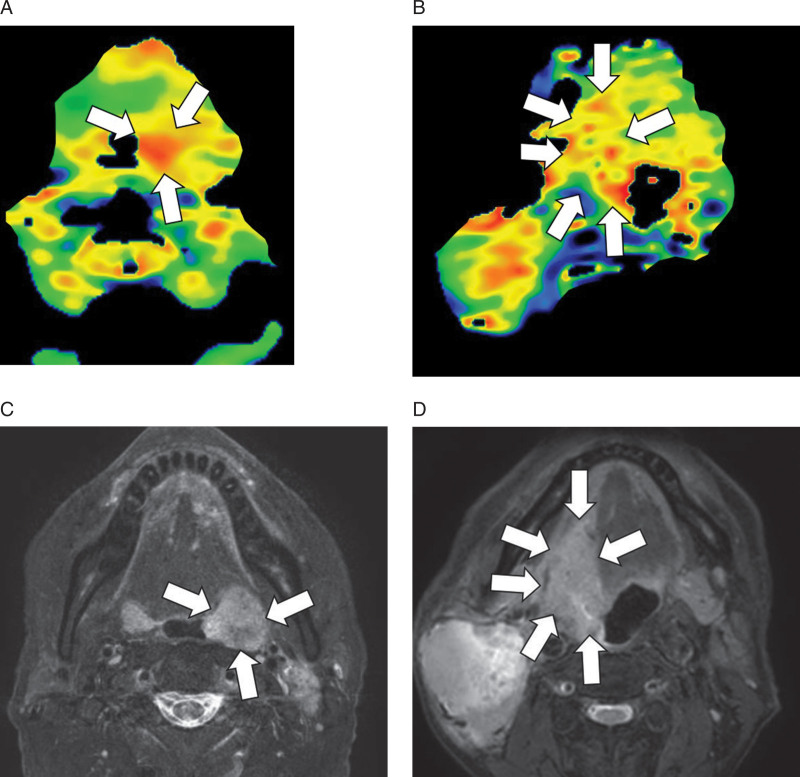
Representative cases of HPV positive and negative oropharyngeal SCC. APT image of HPV-positive oropharyngeal SCC showed a homogeneous intratumoral signal in the tumor with a CV of 0.42 (A: arrows). In contrast, an HPV-negative case included both the high and low signal spots in the tumor (B: arrows) with a CV of 0.53; this higher value indicated the intratumoral heterogeneity. Fs-T2W images of HPV-positive (C: arrows) and -negative cases (D: arrows) are presented as references.

In all patents, all parameter calculation was successfully performed. Details of each parameter value between HPV-positive and -negative oropharyngeal SCCs are presented in Table 2 and Fig. 2. The value of APT CV in the HPV-positive oropharyngeal SCC group (0.43 ± 0.04) was significantly lower than that in the -negative group (0.48 ± 0.04) (*P* = .01). By contrast, there was no significant difference in APT mean (*P* = .82) or APT SD (*P* = .13) between HPV-positive and -negative oropharyngeal SCC groups. The ROC curve analysis of APT CV showed a sensitivity of 0.75, specificity of 0.8, PPV of 0.75, NPV of 0.8, accuracy of 0.77 and AUC of 0.8 with the cut-off value of 0.45. ROC curves are shown in Fig. 3. Representative cases of HPV positive and negative status are shown in Fig. 4.

**Table 2:** Details of all parameters.

	HPV positive (n = 16)	HPV negative (n = 15)	*P* value
APT mean	2.65 ± 0.48	2.68 ± 0.43	0.82
APT SD	1.15 ± 0.24	1.3 ± 0.25	0.11
APT CV	0.43 ± 0.04	0.48 ± 0.04	0.004

Data are mean ± standard deviation.

APT = amide proton transfer, SD = standard deviation, CV = coefficient of variation, HPV = human papillomavirus.

## 4. Discussion

Our results suggested that the mean APT signals of HPV-positive and -negative oropharyngeal SCC were not significantly different; in contrast, the CV of the intratumoral APT signal was significantly lower in HPV-positive oropharyngeal SCCs than in -negative cases; in other words, the APT signal in HPV-negative SCC was significantly more heterogeneous. APT signal information might be a useful tool for the assessment of patients with oropharyngeal SCC. HPV status is usually determined by histopathological p16 immunostaining for its simplicity.^[20]^ However, insufficient materials in biopsy tissue sample sometimes cause a delay in the determination of HPV status. Moreover, histological diagnosis is sometimes difficult in patients with hemorrhagic diathesis. In such cases, the findings of APT imaging might be supportive information in the determination of HPV status. In particular, APT imaging is completely noninvasive, obviating the use of contrast agent; therefore, this technique can contribute to better patient care in daily clinical practice.

In recent years, differences on imaging between patients with HPV-positive and -negative oropharyngeal SCC have been investigated using several major modalities. On CT, HPV-positive oropharyngeal SCCs were reported to have exophytic growth with well-defined margins, in contrast, HPV-negative tumors tended to show adjacent muscle invasion and irregular margins.^[2]^ Several previous studies have reported differences of intratumoral characteristics between HPV-positive and -negative SCCs in various modalities, including standardized Uptake Value (SUV) uptake in fluorodeoxyglucose-positron emission tomography (FDG-PET),^[21]^ image density in CT,^[22–24]^ and signal intensity in MRI.^[25–27]^ These reports found HPV-negative oropharyngeal SCCs to have more intratumoral heterogeneity than HPV-positive cases. In addition, another report described that the ADC values calculated from DWI appeared as a more heterogeneous histogram in HPV-negative oropharyngeal SCCs than HPV-positive cases.^[7]^ The result of the present study indicated the intratumoral heterogeneity of APT signals in HPV-negative oropharyngeal SCCs. This result was consistent with results from these previous studies.

In terms of microstructure, HPV-positive oropharyngeal SCCs have been characterized as well-organized cell clusters with scant interstitial space.^[7]^ In contrast, HPV-negative oropharyngeal SCCs are characterized as including various grades of intratumoral differentiation and the presence of focal hypoxia; this might be responsible for the more heterogeneous intratumoral characteristics.^[26]^ Such heterogeneous factors in HPV-negative tumors likely affect the intratumoral metabolism of tumor proteins/peptides and thus result in the heterogeneity of APT imaging signals. Thus, signal heterogeneity in APT imaging, likely reflecting the heterogeneous protein metabolism of the tumor, joins previously reported imaging information such as conventional CT/MRI, MR-DWI and FDG-PET as a useful indicator of tumor HPV status in oropharyngeal SCCs. The diagnostic performance obtained from APT imaging was revealed to have an AUC of 0.8 in the present study while those from the abovementioned studies were around 0.7–0.85,^[23,26,27]^ suggesting the methods have roughly equal accuracy. The investigation of a still more accurate method, such as the combined use of these parameters, should be performed to clarify the most accurate tool for the assessment of HPV status in oropharyngeal SCCs. In clinical practice, histopathological p16 immunostaining is used as the standard method for the pretreatment diagnosis of HPV status using biopsy tissue. However, a previous review article found that the diagnosis of HPV status by p16 immunostaining occasionally results in failure, with a sensitivity of around 0.75–1.0 and specificity of around 0.8–1.0; moreover, a few studies introduced in this review article have found even lower low specificity, below 0.7.^[28]^ From this point of view, we speculated that a more supportive tool using radiological imaging may be needed for more accurate diagnosis of HPV status.

The current study has several limitations. First, the number of patients was small because this study was performed at a single institution and used a specific sequence (i.e., APT imaging) not included in routine clinical practice. Therefore, our results should be treated as preliminary. Second, we assessed the relation of APT imaging data and HPV status only; assessment of other important factors such as the predictive value of treatment outcome was not performed. Because the patient treatment strategy in the present study cohorts was not uniform, further study will be needed.

## 5. Conclusions

Intratumoral heterogeneity of APT imaging in patients with oropharyngeal SCC was revealed to significantly depend on HPV status. This technique might be a useful supportive tool for assessment of patients with oropharyngeal SCC.

### Author contributions

Conceptualization: Noriyuki Fujima, Kohsuke Kudo

Data curation: Seijiro Hamada, Takayoshi Suzuki, Nayuta Tsushima, Satoshi Kano, Akihiro Homma

Formal analysis: Noriyuki Fujima, Masami Yoneyama, Junichi Nakagawa

Funding acquisition: Noriyuki Fujima

Investigation: Noriyuki Fujima, Yukie Shimizu, Junichi Nakagawa, Hiroyuki Kameda, Taisuke Harada, Seijiro Hamada, Takayoshi Suzuki, Nayuta Tsushima, Satoshi Kano, Akihiro Homma

Methodology: Noriyuki Fujima, Masami Yoneyama

Software: Masami Yoneyama

Supervision: Kohsuke Kudo

Validation: Yukie Shimizu, Hiroyuki Kameda

Visualization: Noriyuki Fujima, Yukie Shimizu, Masami Yoneyama

Writing - original draft: Noriyuki Fujima

Writing - review & editing: Yukie Shimizu, Masami Yoneyama, Junichi Nakagawa, Hiroyuki Kameda, Taisuke Harada, Seijiro Hamada, Takayoshi Suzuki, Nayuta Tsushima, Satoshi Kano, Akihiro Homma, Kohsuke Kudo
